# The Diuretic Action of Weak and Strong Alcoholic Beverages in Elderly Men: A Randomized Diet-Controlled Crossover Trial

**DOI:** 10.3390/nu9070660

**Published:** 2017-06-28

**Authors:** Kristel C. M. M. Polhuis, Annemarthe H. C. Wijnen, Aafje Sierksma, Wim Calame, Michael Tieland

**Affiliations:** 1 The Dutch Beer Institute, Wageningen 6701, The Netherlands; wijnen@kennisinstituutbier.nl (A.H.C.W.); sierksma@kennisinstituutbier.nl (A.S.); 2 Division of Health and Society, Wageningen University, Wageningen 6706, The Netherlands; 3 StatistiCal B.V., 2241 MN Wassenaar, The Netherlands; w.calame@kpnplanet.nl; 4 Faculty of Sports and Nutrition, Amsterdam University of applied sciences, Amsterdam 1097, The Netherlands; m.tieland@hva.nl; 5 Division of Human Nutrition, Wageningen University, Wageningen 6700, The Netherlands

**Keywords:** hydration, dehydration, moderate alcohol consumption, beer, wine, spirits

## Abstract

With ageing, there is a greater risk of dehydration. This study investigated the diuretic effect of alcoholic beverages varying in alcohol concentration in elderly men. Three alcoholic beverages (beer (AB), wine (AW), and spirits (S)) and their non-alcoholic counterparts (non-alcoholic beer (NAB), non-alcoholic wine (NAW), and water (W)) were tested in a diet-controlled randomized crossover trial. For the alcoholic beverages, alcohol intake equaled a moderate amount of 30 g. An equal volume of beverage was given for the non-alcoholic counterpart. After consumption, the urine output was collected every hour for 4 h and the total 24 h urine output was measured. AW and S resulted in a higher cumulative urine output compared to NAW and W during the first 4 h (effect size: 0.25 mL *p* < 0.003, effect size: 0.18 mL, *p* < 0.001, respectively), but not after the 24h urine collection (*p* > 0.40, *p* > 0.10). AB and NAB did not differ at any time point (effect size: −0.02 mL *p* > 0.70). For urine osmolality, and the sodium and potassium concentration, the findings were in line. In conclusion, only moderate amounts of stronger alcoholic beverages, such as wine and spirits, resulted in a short and small diuretic effect in elderly men.

## 1. Introduction

The ageing population is increasing rapidly: by 2030, more than 25% of the population in Europe and Northern America will be over the age of 60 [1]. Older adults are at a greater risk of dehydration due to a lack of thirst, changes in body mass, changes in the water and sodium balance of the body, and a declining renal function [2,3]. With ageing, the risk of chronic disease increases, which may subsequently increase the intake of different sorts of medication. Polypharmacy may also influence hydration status [3]. Dehydration is associated with poor health outcomes. It may increase the risk of thromboembolic complications, infectious diseases, and obstipation [2,4]. Furthermore, a chronic low water intake may be associated with urolithiasis [5,6].

A large proportion of the elderly consume alcohol in moderation (i.e., two to three drinks per day) [7]. Alcohol consumption is known to increase the urine output, which could interfere with normal hydration [8]. Therefore, some institutions recommend in their guidelines on hydration not to drink strong alcoholic beverages, especially in the case of elderly people [9,10].

However, no consistent theory exists on the extent of diuresis due to alcohol consumption. A study in rats from 1968 has shown that the diuretic response to alcohol is directly related to alcoholic concentration [11]. This would suggest that strong (distilled) alcoholic beverages may provoke more dehydration than weak alcoholic beverages. On the other hand, Eggleton concluded in 1942 that an additional 100 mL of urine would be produced for each 10 g of alcohol ingested, though this estimate is based on the data of only one subject, and the alcohol concentration is not mentioned [12]. The US Institute of Medicine concluded in 2005 [13] that the effect of alcohol consumption on increasing urine secretion is transient, and would not result in appreciable fluid losses. This seems to be supported by a recent study on the beverage hydration index [14]. According to this study, there were no differences in the cumulative urine output between lager and still-water up to 4 h after consumption. Only a few studies investigated the effect of stronger alcoholic beverages on hydration status in humans and these suggest that strong (distilled) alcoholic beverages might provoke dehydration [15]. Nevertheless, experimental studies on the diuretic effects of alcohol in the elderly are lacking.

Therefore, the aim of the current study is to examine the diuretic effect of moderate amounts of commercially available weak and strong alcoholic beverages and their non-alcoholic counterparts in elderly men in a normal-life situation. To the best of our knowledge, this is the first study to investigate the net difference in the urine output between beverages varying in alcohol concentration. The outcomes are important for health communication purposes, specifically towards the elderly, who are at an increased risk of dehydration.

## 2. Materials and Methods

### 2.1. Participants

**Figure 1:**
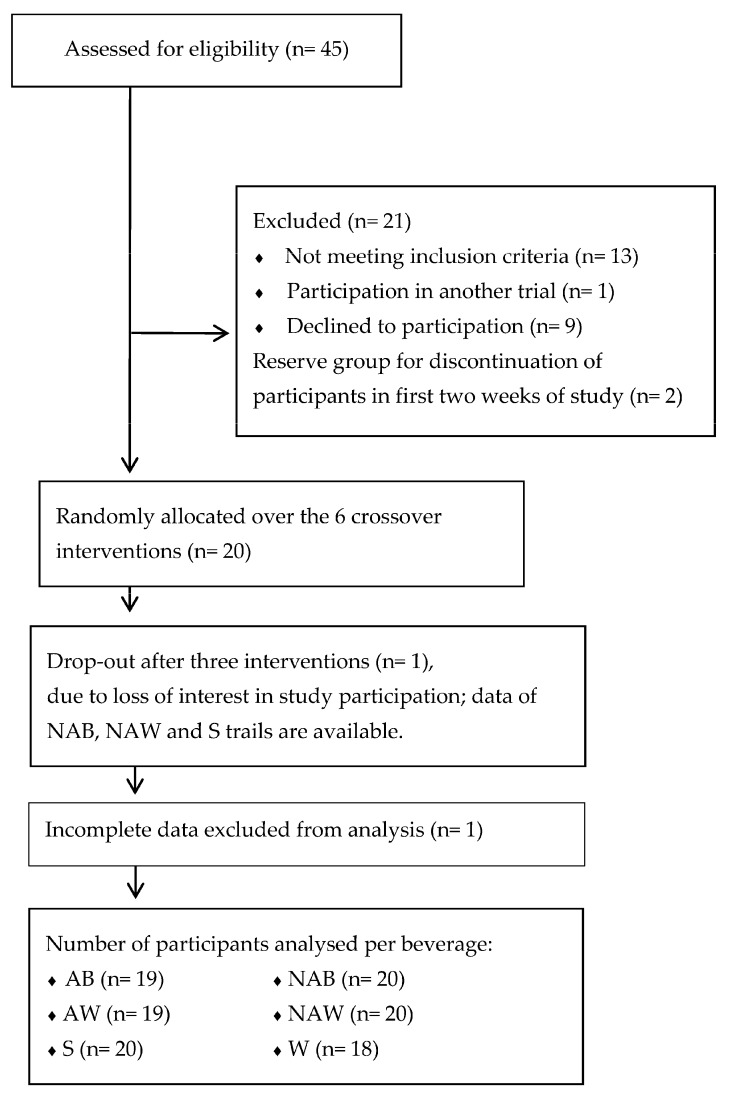
Participant flow through the study. AB: alcoholic beer. NAB: non-alcoholic beer. AW: alcoholic wine. NAW: non-alcoholic wine. S: spirits. W: water.

In total, 20 Dutch men (60–75 years of age) participated in this study (Figure 1). They were recruited in and around Wageningen. The volunteers received information about the study by verbal briefing and in writing. Thereafter, renal function was assessed via measures of plasma creatinine and serum urea levels (normal levels defined as 60–110 mmol/L for creatinine and 2.5–6.4 mmol/L for urea). The volunteers also filled out a screening questionnaire to assess whether they met the inclusion criteria: the occasional consumption of alcoholic beverages (<21 standard alcoholic drinks (10 g of alcohol per drink) per week), no (family) history of alcoholism, and no use of drugs or medication that could interfere with diuresis. When volunteers fulfilled the inclusion criteria and had normal blood levels of creatinine and urea, they were found eligible to participate in the study. All participants gave their informed consent for inclusion before they participated in the study. The study was conducted in accordance with the Declaration of Helsinki, and the protocol was approved by the Medical Ethical Committee of Wageningen University (ethical approval code: NL49923.081.14).

### 2.2. Sample Size Calculation

In total, 13 participants were needed to detect any differences between groups, applying a two-sided evaluation with an alpha of 0.05, a power of 0.80, and an effect size of 1.67 mL. This was based on a previous cross-over hydration study [8] in which the cumulative urine output after beer consumption was compared to the cumulative urine output with non-alcoholic beer up to 4 h after ingestion. However, in the present study, a lower amount of alcohol is tested, and the period of urine collection is longer (24 h). Therefore, we decided to increase the sample size to 20 participants.

### 2.3. Study Design

The participants entered a randomized diet-controlled crossover trial consisting of six interventions, and a preliminary screening visit. During the interventions, they were randomly exposed to: beer (AB) (Amstel^®^ pilsner, Heineken, Zoeterwoude, The Netherlands); non-alcoholic beer (NAB) (Amstel^®^ 0.0, Heineken, Zoeterwoude, The Netherlands); red wine (AW) (AH Merlot, Albert Heijn, Zaandam, The Netherlands); non-alcoholic red wine (NAW) (AH non-alcoholic Merlot, Albert Heijn, Zaandam, The Netherlands); spirits (S) (Bols jenever, Lucas Bols N.V., Amsterdam, The Netherlands); or (tap) water (W). Each intervention was separated by a period of at least seven days. The nutritional composition of the beverages is shown in Table 1. An amount equal to 30 g of alcohol was supplied during the AB, AW, and S trials. The WHO defines acceptable levels of drinking for men as ≤30 g alcohol per day [16]. An equal volume of beverage was given for the non-alcoholic counterpart.

### 2.4. Study Protocol

The participants were asked to maintain their normal dietary habits and exercise pattern and to refrain from alcoholic beverages and caffeinated drinks or foods, including tea, coffee, and chocolate, after 6 PM the day before each of the six trial days. They were, however, instructed to drink a 500 mL bottle of water on that evening to assure a similar hydration state before each intervention. On each trial day, a standard total diet was supplied to exclude dietary confounding, and to have control over the total fluid intake (2.5 L fluid in total, of which 1.5 L is from drinks). The provided diet had three different levels of energy intake per trial day, depending on the body weight of the subjects (Table 2). The participants were not allowed to eat or drink anything but the food supplied. Deviations from the protocol, adverse effects, and other details on lifestyle and wellbeing were registered by the participants in the diary that they received at the start of the study. During each test session, participants collected their urine for 24 h, starting after the first-morning urine sample. The participants consumed all foods and drinks at home, except for lunch, which was served at Wageningen University. The participants were asked to use the same mode of transportation to the research facilities and back home each trial day. After arriving at the research facilities, the same lunch was served every trial day and consisted of lasagna (NV FreshMeals SA, Waarschoot, Belgium). During lunch, the test beverages were consumed (on top of the standard daily 2.5 L fluid intake). Each beverage was consumed in three equal portions during the first half hour of lunch (AB or NAB: 250 mL per portion; AW or NAW: 93 mL per portion; S or W: 36 mL per portion). The room temperature was kept constant. The participants were not blinded for treatment because taste and color differences were apparent with these beverages. Only commercially available beverages were used in this study. After lunch (*t* = 0 h), the participants remained rested (seated activities: working, reading) for a 4 h observation phase. At the end of each hour, the urine output was collected (*t* = 1, 2, 3, 4 h in the observation phase). At the end of the observation phase (4 h), the participants that consumed an alcoholic beverage took a breath test. The Breath Alcohol Content (BrAC) was measured (measure range: 0 to 500 BrAC %, Alcotest 6510, Dräger Safety AG & Co KGaA, Lübeck, Germany) to verify if they could go home safely (when BrAC was ≤0.22 mg L^−1^ of expired air). At home, the participants continued to collect urine until the next morning. During this period, participants could void whenever they needed to. Dietary control was not monitored at home. Participants were asked to report deviations from the instructed diet.

### 2.5. Randomization

An independent scientist who was not involved in the study took care of the randomization of the participants and treatment allocation. Block randomization was performed with SAS, v9.3 (Cary, NC, USA). We stratified the data by the sequence of the beverage. No confounding factors were taking into account in the randomization because all participants received all beverages. No period nor carry-over effect was expected.

### 2.6. Study Outcome

The main study outcome was the difference in the cumulative urine output over both 4 h and 24 h between the three alcoholic beverages and their non-alcoholic counterparts (AB vs. NAB, AW vs. NAW, S vs. W), and the differences in urine output among the different types of alcoholic beverages (∆BEER: AB minus NA, ∆WINE: AW minus NAW, ∆SPIRIT: S minus W). Secondary outcomes were differences in urinary osmolality, and urine sodium and potassium concentration.

### 2.7. Urine Collection and Analysis

All urine samples were accurately weighed to the nearest gram on a calibrated scale (Sartorius 1203 MP, Sartorius AG, Göttingen, Germany). An aliquot (5 mL) from each time-point was stored at −20 °C until further analysis. Urine osmolality was measured using freezing-point depression (Osmomat 030, automatic cryoscopic osmometer, Gonotec, Berlin, Germany). Sodium and potassium concentrations were measured by V-Lyte IMT (Dimension Vista^®^ 1500, Siemens Healthcare Global, Erlangen, Germany).

### 2.8. Statistical Analysis

Data were checked for normality by applying the Shapiro-Wilk test and were presented as the mean ± standard deviation (±SD) when normally distributed. Baseline differences in body mass and baseline urine osmolality between the trials were assessed with One-way Repeated Measures Analysis of Variance (ANOVA). Stepwise non-linear General Estimating Equations (GEE) applying repeated measures were used to identify the overall differences in the cumulative urine output, osmolality, sodium, and potassium, as well as the paired differences in the cumulative urine output between AB and NAB, AW and NAW, and S and W over time between the various beverages. The same statistical technique was used to determine at which time points the differences in urine output, osmolality, sodium, and potassium were significantly different between the various beverages. Outcomes were adjusted for confounders: baseline value, age, gender, and BMI. Time was included as both a linear and non-linear parameter. With respect to the paired differences in the cumulative urine output between AB and NAB, AW and NAW, and S and W, the non-absolute difference was applied as a dependent parameter, showing both a positive and negative outcome.

All statistical analyses were carried out using Stata, version 12 (StataCorp, College Station, TX, USA) and GraphPad Prism version 6 (La Jolla, CA, USA). Graphics were performed using GraphPad Prism. A *p*-value below 0.05 was considered statistically significant.

## 3. Results

### 3.1. Participants

In total, we screened 46 men and included 20 men, of which one withdrew from the study because of a treatment-unrelated cause (Figure 1). Participant characteristics are displayed in Table 3. The median age was 69 (65, 75) years. Body mass averaged 77.7 ± 9.9 kg/body weight and did not differ over the trials (*F* = 1.468, *p* > 0.20). The participants had a similar state of hydration at the beginning of each test session: AB: 334 ± 121 mOsmol/kg, NAB: 434 ± 195 mOsmol/kg, AW: 364 ± 135 mOsmol/kg, NAW: 394 ± 141 mOsmol/kg, S: 414 ± 168 mOsmol/kg, and W: 404 ± 163 mOsmol/kg (*F* = 2.403, *p* > 0.07).

### 3.2. Urine Output

#### 3.2.1. Differences between the Alcoholic Beverages and Their Non-Alcoholic Counterparts

**Figure 2:**
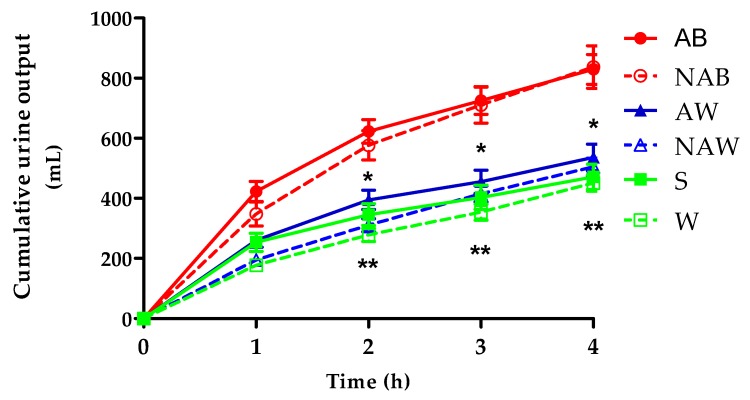
Cumulative urine output over the first 4 h (mean ± standard error of the mean (SEM); *n* ≥ 18). * Significant difference (determined via stepwise General Estimating Equations (GEE) with time as independent parameter) between AW and NAW; *p* < 0.003; ** Significant difference (determined via stepwise GEE with time as independent parameter) between S and W; *p* < 0.003.

Figure 2 displays the relative change in the cumulative urine output per trial over the first 4 h. Table 4 shows the relative change in the cumulative urine output over the first 4 h and over a total of 24 h. During the first 4 h, significant differences were present between AW and NAW (effect size: 0.25 mL, *p* < 0.03), and between S and W (effect size: 0.18 mL, *p* < 0.001). AW resulted in a significantly higher cumulative urine output compared to NAW from 2 h onwards (*p* < 0.003). S increased the urine output compared to W from 2 h onwards (*p* < 0.003). The cumulative urine output between AB and NAB did not significantly differ at any time point (*p* > 0.70), but was significantly higher than the other beverages at all time points (*p* < 0.005). At 24 h, no significant differences were found between the alcoholic and non-alcoholic variants (AB vs. NAB: *p* > 0.50; AW vs. NAW: *p* > 0.40; S vs. W: *p* > 0.10). Time, in either a linear or quadratic configuration, was always a significant confounder over the total 24 h period (*p* < 0.001).

#### 3.2.2. The Difference in Cumulative Urine Output among the Different Types of Alcoholic Beverages

Table 4 displays the three delta’s (∆BEER, ∆WINE, and ∆SPIRITS) of the difference in the cumulative urine output over time. ∆BEER (i.e., urine output AB minus urine output NAB) was not significantly different from ∆WINE (i.e., urine output AW minus urine output NAW) (*p* > 0.10) and ∆SPIRITS (i.e., urine output S minus urine output W) (*p* > 0.10). ∆WINE and ∆SPIRITS were also not significantly different (*p* > 0.80).

### 3.3 Urine Analysis

#### 3.3.1. Urine Osmolality

Table 5 displays the urine osmolality over time. In general, urine osmolality decreased during the first 2 h and increased thereafter. Except for NAB, the urine osmolality was higher than the baseline at 4 h. No significant differences were found between the alcoholic beverages and their non-alcoholic counterparts (AB vs. NAB: *p* > 0.10; AW vs. NAW: *p* > 0.07; S vs. W: *p* > 0.70). The difference between AW vs. NAW approached, but did not reach, significance. The time-dependency analysis shows that the urine osmolality of AW vs. NAW was only significant up to 3 h (*p* < 0.01). The urine osmolality of AB and NAB was significantly lower than the other beverages at all time points (*p* < 0.01). At 24 h, no significant differences were found in urine osmolality between the alcoholic beverages and their non-alcoholic counterparts (AB vs. NAB: *p* > 0.20; AW vs. NAW: *p* > 0.20; S vs. W: *p* > 0.90). Time was a significant confounder over the total 24 h period (*p* < 0.001).

#### 3.3.2. Sodium Concentration

Table 5 displays the urine sodium concentration over time. Except for NAW, the sodium concentration initially decreased at 1 h. Thereafter, the sodium concentration increased for all beverages. At 4 h, the sodium concentration of all beverages was higher than the baseline. A significant difference was found between one beverage and its non-alcoholic counterpart: AW and NAW (*p* < 0.001). The sodium concentration of AW and NAW significantly differed at all time points (*p* < 0.008). The sodium concentration of S and W was significantly different up to 2 h (*p* < 0.05). Sodium concentration was not significantly different for AB and NAB at any time point (*p* > 0.10). At 24 h, the difference between AW and NAW was still significant (*p* < 0.001). The differences between AB and NAB and between S and W were not significant (both *p* > 0.10). Time and the starting value were significant confounding factors (*p* < 0.001).

#### 3.3.3. Potassium Concentration

Table 5 displays the urine potassium concentration over time. Except for AB, the potassium concentration is higher after 4 h compared to the baseline. Significant differences are found between two alcoholic beverages and their non-alcoholic counterparts: AW and NAW (*p* < 0.001) and S and W (*p* < 0.001). The differences were significant at all time points (AW and NAW: *p* < 0.003; S and W: *p* < 0.007). No difference was found between AB and NAB at any time point. (*p* > 0.80). At 24 h, the differences between AW and NAW, and between S and W, were still significant (AW vs. NAW: *p* < 0.001; S vs. W *p* < 0.001). Time and the starting value were significant confounders (*p* < 0.001).

## 4. Discussion

The present study is the first to test the diuretic effect of moderate amounts of weak and strong alcoholic beverages in euhydrated elderly men. The main finding of this study is that moderate amounts of stronger alcoholic beverages (≥13.5%; wine and distilled beverages) provoke a short-term and small diuretic effect, whereas weaker alcoholic beverages, such as beer (5%), do not. Significant differences in the cumulative urine output, osmolality, and sodium and potassium concentration were only present between AW and NAW, and between S and W, during the first 4 h after intake. No significant differences were found between AB and NAB for the urine output, osmolality, and sodium and potassium concentration at any time point. This may imply that the acute effect of alcohol on the cumulative urine output is directly dependent on the alcohol concentration and not on the net alcohol content.

Research in rats shows that the acute diuretic response to alcohol is related to the alcoholic concentration [11]. This result is also supported by human studies, which demonstrates that beer containing up to 2% alcohol does not influence fluid retention after exercise, but stronger beers (3–5%) do [17,18,19,20]. In the present study, full-strength beer (5%) did not affect hydration, while in the aforementioned studies, the beers with an alcohol content >2% tended to impair rehydration. These contradictive findings can probably be explained by differences in the net amounts of alcohol that were used, and differences in the state of hydration of the subjects. In contrast to the large amounts of alcohol (ranging from 50–120 g alcohol) that was used in aforementioned studies, the present study aimed to reflect the normal-life situation of older adults and therefore used a more moderate amount of 30 g of alcohol. Recent research supports our finding that moderate amounts of full-strength beer (≥4.5%, 20–40 g alcohol) does not affect hydration in dehydrated [20,21] and euhydrated (young) men [14].

Furthermore, differences in the cumulative urine output between AW and NAW, and between S and W, were present from 2 h onwards. Previous studies support these results, showing that differences in the urine output appear only 1–2 h after beverage intake [14,17,18,19,20,21,22]. However, the differences in the cumulative urine output between AW and NAW, and between S and W, were not significant at 24 h. This is in line with a previous study that demonstrated that ethanol (1.2 g/kg) caused a diuretic effect during the first 3 h, but an antidiuretic effect 6 h after intake [23]. Also, studies on other dehydrating beverages, such as caffeinated beverages, show that diuretic effects are only short-term [24,25]. In addition, the differences in the urine output between alcoholic and non-alcoholic beverages after exercise disappear from 4 h onwards [17,22]. Based on this, the relevance of the diuretic effect of moderate alcohol consumption in the real-life situation under normal circumstances, can be questioned.

Although it has been hypothesized that strong (distilled) alcoholic beverages provoke more dehydration than weaker alcoholic beverages, experimental evidence is limited. Therefore, we sought not only to determine whether a higher alcohol concentration causes a stronger diuretic effect, but also whether the differences in the urine output between alcoholic beverages and their non-alcoholic counterparts become larger when the alcohol concentration increases. We found no significant differences between ∆BEER, ∆WINE, and ∆SPIRITS, which implies that the net difference in the cumulative urine output between the alcoholic beverage and the non-alcoholic counterpart does not become larger if the alcohol concentration increases. In other words, when taking the differences in the total fluid volume between AW and S into account, S did not cause a higher cumulative urine output compared to AW. To the best of our knowledge, this is the first study to investigate the net difference in urine output between beverages varying in alcohol concentration (instead of varying alcohol content). More research on this topic is needed.

The urine sodium and potassium concentration were significantly different between AW and NAW, and between S and W. However, urine osmolality was not. Urine osmolality is considered the most sensitive measure of urine indices of hydration status [26,27]. AB and NAB resulted in a significantly lower urine osmolality and higher urine output compared to the other beverage, which indicates a better hydration status. This is most likely due to the larger fluid volume intake with AB and NAB consumption. This finding is also in line with previous research that shows that moderate amounts of low-strength alcoholic beverages can result in a net gain of water [15].

Water requirements are not different for older adults compared to younger individuals. However, because of physiological changes due to ageing, older adults are at a higher risk of dehydration. A large proportion of the elderly consume alcohol in moderation, and, therefore, it is important to test the diuretic effects of moderate amounts of various alcoholic beverages in the elderly. This has not been previously done. Besides, as the diuretic effects are studied in a vulnerable population, it is plausible that the results also apply to younger men, because their body mass, water and sodium balance, and renal function, are, in general, better. However, research is needed to confirm this hypothesis. In addition, recent research suggests a gender difference in the regulation of urine production [28]. The present results should therefore not be directly translated to (elderly) women as their body mass and composition is different and the possible impact of hormones on fluid balance cannot be excluded [28].

The present study has several strengths. First of all, the diet-controlled crossover design minimizes the influence of confounding variates. Secondly, by testing moderate amounts of alcohol in a normal-life situation, the results can easily be translated to the real-life situation. In addition, the study included a 24 h urine measurement which provides more insight into the duration of the effect, and thus the impact in daily life. This is also a limitation of this study as we cannot rule out the incompliance of urine collection and dietary instructions between 4 and 24 h, since this was done at home. However, if urine samples were incomplete, we expect this to have happened randomly, without influencing the outcome of the study. A second limitation comprises the nutritional differences between the alcoholic and control beverages. This could have influenced the rate of gastric emptying and thus the hydration rate during the first 4 h measurements. Eventually, the total amount of fluid would be reflected in the water balance, so we do not expect that this affected the interpretation of the data. In addition, it is important to note that the beverages were not consumed in the fasting state, but together with a meal. We expect that this minimizes the differences in gastric emptying and diuresis compared to the fasting state. We have deliberately chosen commercially available drinks to increase the practical applicability. Future research, however, might consider matching the beverages in terms of the nutritional composition. A final limitation is that no blood measurements were included, and, therefore, we are not able to provide insights on the mechanism underlying the diuretic effects of alcohol. To date, knowledge of the mechanism remains incomplete. In a healthy person, the circulating hormones arginine vasopressin and aldosterone largely determine the produced volume of urine [29]. Arginine vasopressin and aldosterone are regulated by the body’s salt and water balance. The current hypothesis on the diuretic effect of alcohol states that ethanol increases the urine output by suppressing arginine vasopressin release of the posterior pituitary gland independently of blood osmolality [30,31,32]. However, studies measuring arginine vasopressin levels after alcoholic and non-alcoholic beverages did not find differences in arginine vasopressin levels [17,19], suggesting that other mechanisms are involved [22].

## 5. Conclusions

In conclusion, only moderate amounts of stronger alcoholic beverages (≥13.5%) resulted in a temporary diuretic effect compared to their non-alcoholic counterparts. AB and NAB did not differ at any time point in terms of the urine output, osmolality, and sodium and potassium concentration. This suggests that the consumption of moderate amounts of a weak alcoholic beverage such as beer is safe in terms of hydration for elderly men. Taking the results of previous studies into account, it seems that moderate amounts of weak alcoholic beverages do not negatively influence hydration status, but when the alcoholic strength and serving size increases, so does the acute diuretic effect. However, the diuretic effect of stronger alcoholic beverages was small and short-lived, and therefore, the diuretic effect of moderate alcohol consumption—independent of the alcohol concentration—may be transient and thus negligible in euhydrated elderly men. More research is needed to establish whether similar effects apply to women.

## Figures and Tables

**Table 1:** Nutritional composition of the test beverages.

Beverage	Energy (kJ) ^1^	Carbohydrates (g) ^1^	Sodium (mmol/L)	Alcohol (% abv ^2^)
Alcoholic Beer (AB)	170	3	1.1	5.0
Non-Alcoholic Beer (NAB)	101	6	1.3	0.0
Alcoholic Wine (AW)	79	0.2	0.0	13.5
Non-Alcoholic Wine (NAW)	75	4.5	0.0	0.0
Spirits (S)	194	0.0	0.0	35.0
Water (W)	0	0	0.8	0.0

^1^ Presented as 100 g of product; ^2^ abv = alcohol by volume.

**Table 2:** Nutritional composition of the diet.

	Diet A (*n* = 5)	Diet B (*n* = 6)	Diet C (*n* = 9)
Energy (kJ)	7750	8628	9374
Fat (g)	56.1	76.7	86
Protein (g)	66.8	90.2	98.8
Carbohydrates (g)	254.7	236.1	249.8
Dietary fibre (g)	19.3	19.4	21.8
Sodium (mg)	2202	2622	2955

**Table 3:** Baseline characteristics.

Characteristics	Mean ± Standard Deviation (*n* = 20)
Age (year)	69 (65, 75) ^1^
Weight (kg)	77.1 ± 9.8
Body Mass Index (kg/m^2^)	25.1 ± 2.2
Creatinine blood levels (mmol/L)	84.8 ± 8.7
Urea (mmol/L)	6.4 ± 1.2

^1^ Median (minium, maximum).

**Table 4:** Cumulative urine output in mL per beverage (AB, NAB, AW, NAW, S, and W; mean ± standard deviation (SD)) and the difference (∆) in cumulative urine output in mL among the different types of alcoholic beverages (∆BEER, ∆WINE, ∆SPIRITS; mean ± SD) over time.

	Cumulative Urine Output (mL)						Difference in Cumulative Urine Output (mL)		
Hours after Consumption	AB (*n* = 19)	NAB (*n* ≥ 19)	AW (*n* = 19)	NAW (*n* = 20)	S (*n* = 20)	W (*n* = 18)	∆BEER (*n* = 19)	∆WINE (*n* = 19)	∆SPIRITS (*n* = 18)
0	0 ± 0.00	0 ± 0	0 ± 0	0 ± 0	0 ± 0	0 ± 0	0 ± 0	0 ± 0	0 ± 0
1	423 ± 146	347 ± 179	260 ± 100	194 ± 81	253 ± 135	177 ± 74	73 ± 191	71 ± 119	81 ± 150
2	622 ± 170	576 ± 219	394 ± 139 ^1^	310 ± 96 ^1^	345 ± 162 ^2^	277 ± 92 ^2^	28 ± 200	88 ± 158	74 ± 146
3	725 ± 201	710 ± 268	455 ± 170 ^1^	414 ± 119 ^1^	403 ± 180 ^2^	353 ± 113 ^2^	−11 ± 243	40 ± 172	57 ± 157
4	829 ± 217	836 ± 316	536 ± 192 ^1^	504 ± 130 ^1^	471 ± 189 ^2^	450 ± 114 ^2^	−45 ± 276	35 ± 193	31 ± 149
24	2459 ± 252	2435 ± 660	2028 ± 252	2163 ± 297	1846 ± 276	1918 ± 470	−118 ± 356	−110 ± 340	−75 ± 589

^1^ Significant difference (determined via stepwise GEE with time as independent parameter) between AW and S: *p* < 0.003; ^2^ Significant difference (determined via stepwise GEE with time as independent parameter) between S and W: *p* < 0.003.

**Table 5:** Urine osmolality, sodium, and potassium values over time per trial (mean ± SD; *n* ≥ 18).

	Hours after Consumption	AB	NAB	AW	NAW	S	W
Urine Osmolality (mOsmol/kg)	Pre	334 ± 121	434 ± 195	364 ± 135	394 ± 141	414 ± 168	404 ± 163
	1	194 ± 35	184 ± 51	278 ± 110 ^1^	542 ± 255 ^1^	279 ± 158	463 ± 125
	2	319 ± 139	227 ± 44	475 ± 259 ^1^	537 ± 219 ^1^	520 ± 256	585 ± 130
	3	447 ± 228	332 ± 166	585 ± 322 ^1^	519 ± 163 ^1^	734 ± 110	616 ± 73
	4	465 ± 197	411 ± 183	633 ± 215	533 ± 124	769 ± 97	452 ± 137
	24	296 ± 30	278 ± 11	291 ± 51	266 ± 19	366 ± 6	323 ± 228
Urine Na^+^ (mmol/L)	Pre	52 ± 24	58 ± 23	52 ± 24	58 ± 20	59 ± 32	58 ± 27
	1	32 ± 6	32 ± 13	43 ± 16 ^2^	62 ± 26 ^2^	47 ± 20 ^3^	58 ± 29 ^3^
	2	42 ± 17	38 ± 14	58 ± 22 ^2^	77 ± 25 ^2^	65 ± 29 ^3^	767 ± 37 ^3^
	3	62 ± 29	53 ± 21	78 ± 32 ^2^	82 ± 17 ^2^	82 ± 34	84 ± 38
	4	66 ± 27	66 ± 26	85 ± 33 ^2^	93 ± 20 ^2^	90 ± 35	89 ± 31
	24	52 ± 11	51 ± 8	54 ± 12 ^2^	55 ± 14 ^2^	59 ± 13	56 ± 12
Urine K^+^ (mmol/L)	Pre	30 ± 15	41 ± 21	34 ± 18	31 ± 14	36 ± 16	38 ± 19
	1	17 ± 6	23 ± 14	27 ± 17 ^4^	45 ± 23 ^4^	28 ± 18 ^5^	47 ± 18 ^5^
	2	22 ± 12	26 ± 10	35 ± 20 ^4^	55 ± 18 ^4^	37 ± 19 ^5^	54 ± 20 ^5^
	3	32 ± 16	36 ± 16	44 ± 21 ^4^	55 ± 19 ^4^	45 ± 24 ^5^	56 ± 18 ^5^
	4	43 ± 23	39 ± 17	55 ± 22 ^4^	53 ± 17 ^4^	54 ± 22 ^5^	54 ± 21 ^5^
	24	24 ± 5	22 ± 5	29 ± 7 ^4^	22 ± 9 ^4^	28 ± 7 ^5^	23 ± 5 ^5^

^1^ Significant different: *p* < 0.01; ^2^ Significant different: *p* < 0.008; ^3^ Significant different: *p* < 0.05; ^4^ Significant different: *p* < 0.003; ^5^ Significant different: *p* < 0.007.

## Abbreviations

The following abbreviations are used in this manuscript:

AB	Alcoholic beer
NAB	Non-alcoholic beer
AW	Alcoholic wine
NAW	Non-alcoholic wine
S	Spirits
W	Water
% abv	Alcohol by volume
BrAC	Breath alcohol content
∆BEER	Urine output AB minus urine output NAB
∆WINE	Urine output AW minus urine output NAW
∆SPIRIT	Urine output S minus urine output W
